# The Internal Conduit System of the Swine Inverted Lymph Node

**DOI:** 10.3389/fimmu.2022.869384

**Published:** 2022-06-06

**Authors:** Laurence Dubreil, Mireille Ledevin, Caroline Hervet, Déborah Menard, Claire Philippe, François J. Michel, Thibaut Larcher, François Meurens, Nicolas Bertho

**Affiliations:** ^1^ APEX, PAnTher, INRAE, Oniris, Nantes, France; ^2^ BIOEPAR, INRAE, Oniris, Nantes, France; ^3^ INMED - UMR 1249 INSERM - Aix-Marseille Université, Marseille, France; ^4^ Department of Veterinary Microbiology and Immunology, Western College of Veterinary Medicine, University of Saskatchewan, Saskatoon, Canada

**Keywords:** swine (source: MeSH NLM), lymph node (LN), endothelial cell (EC), follicle, B lymphocytes, second harmonic generation (SHG), fluorescence imaging (FLI), whole organ imaging

## Abstract

Lymph nodes (LN) are the crossroad where naïve lymphocytes, peripheral antigens and antigen presenting cells contact together in order to mount an adaptive immune response. For this purpose, LN are highly organized convergent hubs of blood and lymphatic vessels that, in the case of B lymphocytes, lead to the B cell follicles. Herein take place the selection and maturation of B cell clones producing high affinity antibodies directed against various antigens. Whereas the knowledge on the murine and human LN distribution systems have reached an exquisite precision those last years, the organization of the antigens and cells circulation into the inverted porcine LN remains poorly described. Using up to date microscopy tools, we described the complex interconnections between afferent lymphatics and blood vessels, perifollicular macrophages, follicular B cells and efferent blood vessels. We observed that afferent lymphatic sinuses presented an asymmetric Lyve-1 expression similar to the one observed in murine LN, whereas specialized perifollicular sinuses connect the main afferent lymphatic sinus to the B cell follicles. Finally, whereas it was long though that mature B cells egress from the inverted LN in the T cell zone through HEV, our observations are in agreement with mature B cells accessing the efferent blood circulation in the efferent, subcapsular area. This understanding of the inverted porcine LN circuitry will allow a more accurate exploration of swine pathogens interactions with the immune cells inside the LN structures. Moreover, the mix between similarities and differences of porcine inverted LN circuitry with mouse and human normal LN shall enable to better apprehend the functions and malfunctions of normal LN from a new perspective.

## Introduction

Lymph nodes (LN) are the essential headquarters of the adaptive immune response initiation. Upon infection, free or dendritic cell-associated antigens are drained from peripheral tissues to LN through the afferent lymphatics, while naïve blood borne effector T and B lymphocytes reach LN through high endothelial venules (HEV) situated in the LN T cell zone (TZ). Antigen bearing DC and T cells encounter each other in the TZ of the LN. In the same area, naïve B cells are pre-activated upon encounter of their cognate antigen. However, for full activation and to fulfill their complex maturation process, pre-activated B lymphocytes must then migrate into the B cell follicle, where follicular helper T cells (Tfh) and follicular DC (FDC) accompany the B cell maturation. This process encompasses the centroblasts step, in which B cells experience B cell receptor hypermutations leading to variations of antibody affinity, and the emergence of B cells expressing antibodies with higher affinity to their cognate antigen. These high-affinity antibodies-expressing B cells are selected by FDC/Tfh interactions. Selected B cell then mature to centrocyte, plasmablast and finally plasmocyte [for review see (1)]. This follicular-maturation process is mandatory for the development of B cell-clones producing high-affinity neutralizing antibodies, which are an indispensable weapon of the anti-microbial responses.

B cell maturation requires the presence of the cognate-antigen, and thus the translocation of soluble antigens from afferent lymph to B cell follicle. In mouse, this process is taken in charge by subscapular sinus macrophages (SCS Mθ) (2, 3). In mouse, LN circuitry is composed of sinuses and venules constituted by, respectively, lymphatic (LEC) and blood (BEC) endothelial cells. In addition, the LN core is served by collagen conduits surrounded by fibroblast reticular cells (FRC) (4, 5) that connect subcapsular sinus to high endothelial venules (HEV), [for review see (6)].

To escape the adaptive immune response, pathogens are known to inhibit several initiation steps occurring in the LN. For instance, in mouse, *Staphylococcus aureus*, *Streptococcus spp*, influenza A virus and vaccinia virus alter the SCS Mθ/B cell follicle interface (7). In swine, at least two economically important pathogens, the porcine circovirus 2 (PCV2) and the porcine reproductive and respiratory syndrome virus (PRRSV) escape the adaptive immune response by altering the B cell development (8). One hypothesis among others is that these alterations occur in the LN, although the exact mechanisms involved remained unknown. A better understanding of the porcine LN should facilitate the exploration of PCV2 and PRRSV actions on the initiation of the immune response.

Porcine LN possess the peculiarity to be ‘inverted’ compared to murine or human LN (9, 10). For an intelligible scheme of this inverted structure please refer to the Figure 2 of reference (11). In swine the afferent lymph diffuses from the center to the periphery in a centrifugal path, conversely to murine and human LN which present a centripetal lymph flow. This lead to the positioning of the porcine B cell follicles in the depth of the LN. Moreover, mature T and B lymphocytes directly exit from the LN through blood vessels, leading to the intriguing hypothesis that in porcine LN, naïve and mature lymphocytes use the same HEV to respectively enter and exit the LN (12).

In a previous study (13) we defined three macrophage populations of the porcine inverted LN: i) the peri-follicular macrophages (pfMθ) though to be the porcine counterpart of the murine SCS Mθ because of their location in contact with the B cell follicle, ii) the cord Mθ similar to the murine medullary cord Mθ and iii) the efferent Mθ (effMθ), situated at the periphery, before the exit of the porcine LN, and equivalent to murine medullary sinus Mθ. We also described the follicular B cells maturation steps.

Hither, using a fluorescent microscopy descriptive approaches we revisited the previous studies dating from the 80s (9, 10, 14–19) by investigating the potential intra-nodal distribution routes of naïve lymphocytes and peripheral antigens as well as the route of exit of antigen-activated mature B lymphocytes.

## Material and Methods

### Collect, Freezing, Cutting of the LN

Four-month-old healthy conventional Duroc pigs were euthanized and necropsied. One or two tracheobronchial lymph nodes were collected on 5 different animals and snap-frozen in nitrogen cooled isopentane. Frozen samples were cut into 10-μm-thick serial coronary sections for Hemalun-Eosin-Saffran routine staining or stored at -80°C for further immunohistochemistry analysis.

All the animal experiments were authorized by the French Ministry for Research (authorization no. 2020062915381908/APAFIS 26250v3) and approved by the Pays de Loire ethics committee.

### Immunohistochemistry Labelling

Frozen LN sections were thawed at room temperature (RT) and then fixed and permeabilized in cold (-20°C) 1:1 acetone/methanol (V/V) for 20 minutes (min) in the freezer. The fixed/permeabilized sections were then washed in PBS/0.5% Tween 80 (PBS/Tween), and incubated in 50 mM NH_4_Cl in order to decrease the tissue autofluorescence background. After washing in PBS/Tween, sections were saturated using blocking buffer (PBS/Tween, 5% swine serum and 5% donkey serum) for 30 min at 4°C and then stained by incubating with different combinations of primary antibodies described in **Table 1**, overnight at 4°C in a humidity chamber. After PBS/Tween washing, secondary antibodies (anti-mouse IgG1-Alexa Fluor 488, anti-mouse IgG2a-Alexa Fluor 488, anti-rat Alexa Fluor 488, anti-rabbit Alexa Fluor 555, anti-mouse IgG2a-Alexa Fluor 555, anti-mouse IgG1-Alexa Fluor 555, anti- mouse IgG1-Alexa Fluor 647, anti-mouse IgG2b-Alexa Fluor 647, anti-rat Alexa Fluor 647, all from Thermofisher Scientific, Waltham, Massachusetts, USA) related to the combination of primary antibodies, and diluted 1/200 in blocking buffer were added for 1 hour (h) at 4°C. When using 2 mouse IgG1 antibodies on the same slide, one of the IgG1 directly coupled to a fluorochrome (anti-Ki67 Alexa Fluor 555, anti-CD79α Alexa Fluor 647 or anti-CD21-FITC) was added for 1 h at 4°C as third staining step following the secondary-coupled antibodies. Between the secondary and the IgG1 fluorochrome-coupled antibodies, the isotype IgG1 control (1/20 in blocking buffer) was added as saturation step. After the last antibody incubation step, sections were washed in PBS/Tween, fixed in 4% paraformaldehyde and stained with 2 µg/mL 4′,6-diamidino-2-phenylindole (DAPI, Sigma-Aldrich, St. Louis, MO, USA), washed in PBS/Tween and mounted in Mowiol 4-88 mounting medium (Sigma-Aldrich, St Quentin Fallavier, France).

**Table 1:** Antibodies used.

antigen	clone	species	isotype	fluo	dilut°	supplier
**Bcl6**	K11291	mouse	IgG1	none	1/100	BD Pharmingen
**Blimp-1**	3H2-E8	mouse	IgG1	none	1/100	Thermofisher
**CD21**	B-ly4	mouse	IgG1	none	1/100	BD Pharmingen
**CD21**	B-ly4	mouse	IgG1	FITC	1/10	BD Pharmingen
**CD31**	LCI-4	mouse	IgG1	none	1/100	BIORAD
**CD79α**	HM57	mouse	IgG1	AF647	1/10	Thermofisher
**FRC**	ER-TR7	mouse	IgG2a	none	1/100	BIORAD
**Ki67**	B56	mouse	IgG1	AF555	1/10	BD Pharmingen
**veCadherin**	polycl	rabbit	IgG	none	1/100	Santa Cruz
**Lyve-1**	polycl	rabbit	/	none	1/100	Abcam
**Pax5**	1H9	rat	IgG2a	none	1/100	Thermofisher
**CD8a**	PT81B	mouse	IgG2b	none	1/50	WSU
**CD11c**	3A8	mouse	IgG1	none	1/100	I. Schwartz (INRAE, Jouy)
**CD169**	1F1	mouse	IgG2a	none	pure	J. Dominguez (INIA, Madrid)
**IgM**	Pg145A	mouse	IgM	none	1/100	Thermofisher
**MHC-II**	MSA3	mouse	IgG2a	none	1/200	WSU

Images were acquired using a slide scanner (Axio Scan Z1, Zeiss, Jena, Germany) with fluorescence, and brightfield imaging modes (objective used was a Plan Apochromat 10x). Brightfield imaging was performed with LED illumination and Tri-CDD Hitachi camera detection. Fluorescence imaging was performed with i) XCITE LED FIRE illumination, ii) emission Band Pass (EM BP 445/50 (DAPI), EM BP 525/50 (Alexa Fluor 488), EM BP 605/70 (Alexa Fluor 555), EM BP 690/50 (Alexa Fluor 633).

### Whole LN iDISCO Clearing Pretreatment

iDISCO+ clearing protocole (20) and immunolabeling were performed on a small whole lymph node (10x7x3mm^3^) and thick section of a large lymph node (10x5x2 mm^3^). Lymph nodes were fixed in PBS/4% PFA at 4°C, overnight with shaking, then washed in PBS at room temperature (RT) 30 min, 3 times. Sample dehydration was performed using methanol in water increasing concentration serial solutions:20%, 40%, 60%, 80%, 100%, 1 h each. Thereafter, dehydrated samples were washed for 1h in 100% methanol followed by overnight incubation, with shaking, in 66% dichloromethane (Sigma-Aldrich)/33% methanol at RT. Then samples were washed twice in 100% methanol at RT. Sample bleaching was done in chilled fresh 5% H_2_O_2_ in methanol (1 volume 30% H_2_O_2_ to 5 volumes methanol), overnight at 4°C. Then sample rehydration was obtained using methanol in water decreasing concentration serial solutions: 80%, 60%, 40%, 20%, 1 h each at RT and washed twice in PBS 0.2% tritonX100 at RT 1 h.

### Whole LN iDISCO Immunolabeling

The used protocol was adapted from (13). Briefly, samples were incubated for 2 days at 37°C in permeabilization solution (0.2% Triton, 20% DMSO, 0.3M Glycine, 0.05% sodium azide in PBS), then incubated for another 4 days at 37°C blocking solution (0.2% Triton, 10% DMSO, 6% goat serum, 6% swine serum, 0.05% sodium azide in PBS). Primary antibodies (anti-CD169, mouse IgG2a and anti-CD31, mouse IgG1 (**Table 1**) were incubated for 7 days at 37°C in staining solutions (PBS/Tween, 0.01% sodium azide, 10 µg/mL heparin, 10% DMSO, 3% horse serum, 3% swine serum, 100 µg/mL saponin in PBS). Samples were washed in PBS 0.1% tween 80, for 4-5 times, during 2 days at RT. Secondary antibodies [anti-mouse IgG2a-Alexa Fluor 488, and anti-mouse IgG1-Alexa Fluor 555 (**Table 1**)] were incubated in staining solution for 7 days at 37°C. Finally, samples were washed 5 times at RT during 2 days in PBS/tween, 10 µg/mL heparin.

### Whole LN iDISCO Clearing

Immunolabelled sample was dehydrated using methanol in water increasing concentration serial solutions: 20%, 40%, 60%, 80%, 100%, for 1h each step at RT followed by an overnight with 66/33 (v/v) dichloromethane/Methanol. Then, methanol was washed-off from the samples by immersion with shaking in 100% dichloromethane for 20 min at RT repeated two times, and as final step samples were stored in DiBenzyl Ether (DBE, Sigma-Aldrich) at RT.

### Whole LN Acquisitions

Whole cleared LN acquisitions were performed using the UltraMicroscope II (LaVision Biotec, Bielefeld, Germany) coupled with a superK EXTREME (NKT photonics, Southampton, UK) Supercontinuum laser. This laser emits a white light that cover the whole visible spectra. Excitation wavelength was selected with band pass filters (560/40) and converted as a light sheet of 4 µm thickness. Z-stacks (4 µm steps). Fluorescence (emission filter 620/60) were taken with a 2x Olympus objective (NA: 0.5) covered with a 6.5 mm WD cap (geometric aberration corrected, Lavision Biotec) plus a X 0.8 optical zoom (spatial resolution: 3.58 x 3.58 x 4 µm). Confocal microscope was a LSM780 (Zeiss) with an objective lens 20x and dual sequential excitation at 488 nm and 561 nm.

Multiphoton microscope was an A1RMP+ (Nikon Europe B.V., Amsterdam, Netherlands) with NDD GaAsP detectors, an objective lens apochromat 25x MP1300 (NA 1.10, WD 2.0 mm) and dual excitation at 960 nm and 1040 nm (Insight Deepsee laser tunable in the 680-1300 nm range, Spectra Physic, Didcot, UK). The set up used for multiphoton imaging was described in (21). From 960 nm excitation, forwarded Second Harmonic generation was acquired in blue channel using a 400-492nm bandpass filter (SEMRock, Rochester, NY, USA) and 2 Photon fluorescence of Alexa Fluor 488 was acquired in green channel in backward using a 525/50 nm filter (SEMRock). From 1040 nm excitation, 2 Photon fluorescence of Alexa Fluor 555 was acquired in red channel in backward using a 575/25 filter (SEMRock).

### Analysis

2D epifluorescence images were proceeded using ZEN 3.3 blue software (Zeiss). 3D acquired from confocal and multiphoton microscopes were proceeded using NIS-Elements software package. (5.30.03 Nikon Instruments Inc., Nikon Europe B.V., Amsterdam, Netherland). 3D Images acquired from light sheet were proceeded using Imaris software (9.1.2, Oxford Instruments, Abingdon, UK).

Data were analyzed using the GraphPad Prism v5.0 statistical software package (GraphPad Software, La Jolla, CA, USA). Since the data were non normally distributed (normality tested using Shapiro-Wilk test) the non-parametric Mann-Whitney test has been chosen. Significance is depicted (** p<0.01, **** p<0.0001).

## Results

### Location and Orientation of the B Cell Follicles

**Figure 1:**
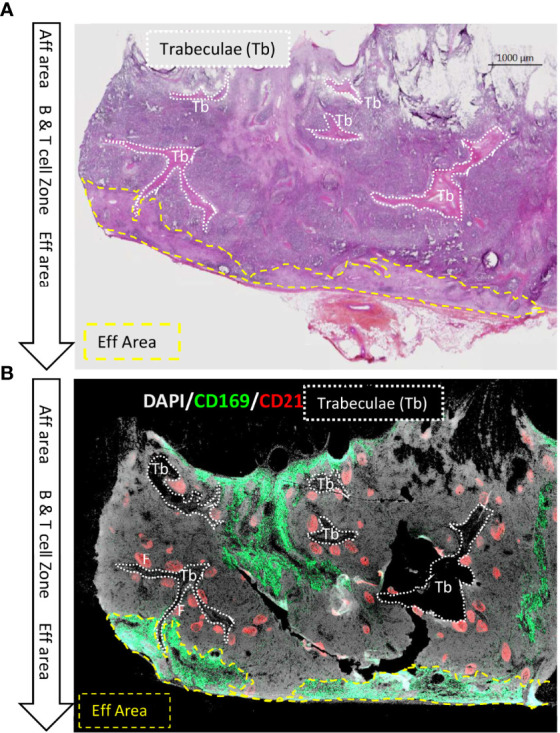
Main features of swine inverted LN in HES and B cells/macrophage immunofluorescent staining on consecutive sections. Consecutive 10 µm thick sections of a tracheobronchial LN were stained using **(A)** HES or **(B)** anti-CD21 (red) and anti-CD169 (green) antibodies and DAPI nuclear staining. Dotted white lines delimited Trabeculae (Tb), dashed yellow lines delimited the efferent area (Eff Area). F: B cell Follicle. Aff area, Afferent area; Eff area, Efferent area. Whole LN pictures are individual images from 10x objective acquisitions, scale bar 1000 µm. Images are representative of 3 LN from different animals.

HES staining of LN sections allowed to identify branched eosinophilic structures characteristic of the previously described trabeculae (**Figure 1A**) (11, 22). Using serial immunolabelled sections for CD169 and CD21, two respective markers of the effMθ and the immature B cell, associated with DAPI-nuclear counterstaining (**Figure 1B**), these trabeculae appeared as dark areas, with a low cell density compared to the rest of the LN parenchyma. Trabeculae are originating from the afferent area and extend to the efferent area. **Figures 1A**, **B** illustrate that porcine LN trabeculae serve the efferent area occupied by CD169^pos^ effMθ (13). Strikingly, all the B cell follicles lied along trabeculae (**Figure 1B**). Because mouse and human LN area are named according to their location in the LN (cortex, paracortex, medulla), porcine immunologists used the same denomination for functionally similar structures, what lead to some confusion, with for instance a porcine LN cortex situated in the center and a medulla in the periphery (11). In order to avoid ambiguity, and to ease the comparison with ‘normal’ LN from mice and humans, here are defined the different porcine LN area with a functional naming: we are naming B cell Zone (BZ) the central area containing the B cell follicles (F), in close contact with the trabeculae. BZ is equivalent to the mouse and human LN cortex. We are naming T cell Zone (TZ) the area that extends from the BZ to the subscapular efferent area containing CD169^+^ effMθ in pigs. TZ is also named paracortex in murine and human LN. The efferent area, or medulla, is peripherally lined by the efferent sinus, which is in a subcapsular location in the inverted swine LN (**Figure 1**).

### Porcine LN Endothelial Cells

**Figure 2:**
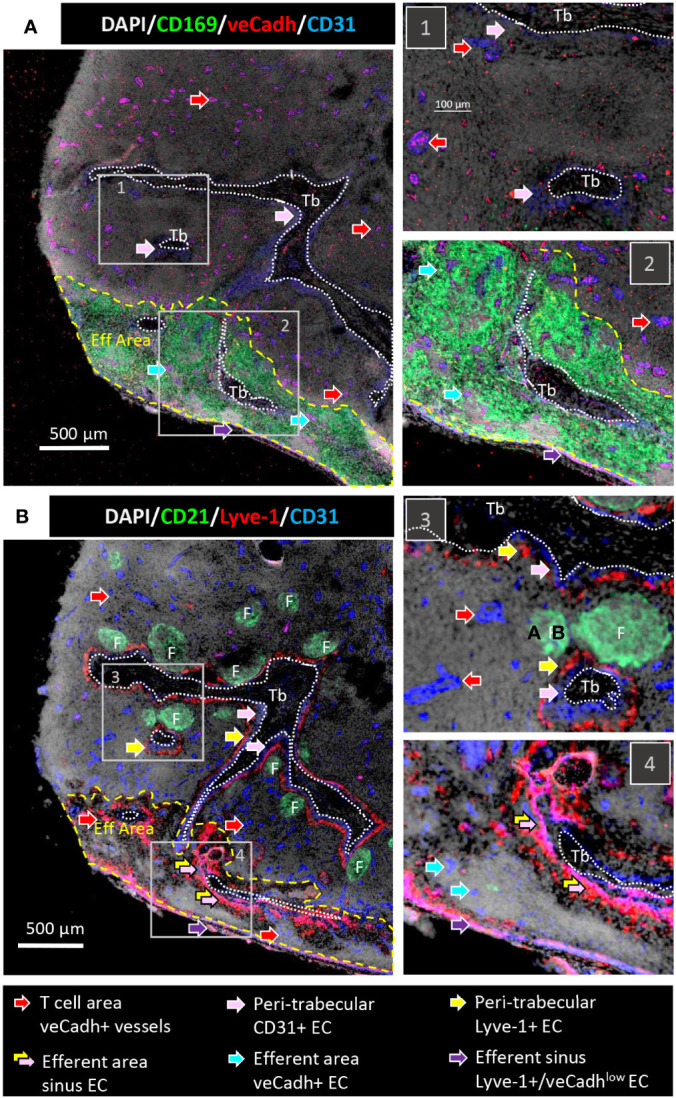
LN vessels and sinuses are delimited by endothelial cells expressing different levels of CD31, veCadherin and Lyve-1. Consecutive 10 µm thick sections from the same LN as in **Figure 1** were stained with **(A)** anti-CD169, anti-veCadherin, anti-CD31 antibodies and DAPI or **(B)** anti-CD21, anti-Lyve-1, anti-CD31 antibodies and DAPI. Tb, Trabeculae; Eff Area, efferent area. F: B cell Follicle. Red arrows: T cell area veCadherin^pos^ vessels, pink arrows: Trabecular CD31^pos^ endothelial cells, yellow arrows: Trabecular Lyve-1^pos^ endothelial cells, double pink/yellow arrows: efferent area sinus endothelial cells, blue arrows: efferent area veCadherin^pos^ vessels, violet arrows: efferent sinus Lyve-1^pos^/veCadherin^pos^ endothelial cells. The right pictures numbered 1 and 2 in A) and 3 and 4 in B) referred to enlargements of the framed regions 1 to 4 on the left large pictures. Whole LN pictures are individual images from 10x objective acquisitions, scale bar 500 µm. Images are representative of 3 LN from different animals.

To identify LN endothelial cells, porcine consecutive LN sections were labelled with an antibody against the pan-endothelial marker CD31. In human, CD31 is more highly expressed on blood endothelial cells (BEC) than on lymphatic endothelial cells (LEC) (23). Two additional endothelial markers presenting more restrictive expressions were used: veCadherin is mainly expressed on BEC but also on some LEC (24), and Lyve-1 is expressed on discrete peripheral LEC in mouse (25) and human (26–28). Since only polyclonal rabbit antibodies were available against both veCadherin and Lyve-1, consecutive sections were used in order to compare their respective locations. Anti-CD31 antibodies labelled vessels, also labelled for veCadherin and localized in the TZ, in agreement with an HEV identity (**Figure 2A**, red arrows). Different, CD31+/veCadherin- cells surrounded trabeculae. These CD31+ cells were themselves contiguous to a Lyve-1 distinctive staining (**Figure 2B**, respectively pink and yellow arrows). These peritrabecular structures were devoid of veCadherin expression (**Figure 2A**). Peritrabecular endothelia presented extensions into the efferent area that were co-labelled for CD31 and Lyve-1 (**Figure 2B**, joined pink/yellow arrows). In the efferent area, CD31+/veCadherin+ vessels can be observed (**Figure 2A**, light blue arrows). Finally, a thin peripheral Lyve-1+/CD31+ labelling was observed, corresponding to the efferent sinus (**Figure 2B**, violet arrow). This efferent sinus expressed low levels of veCadh (**Figure 2A**, violet arrow).

### B Cell Follicles and Trabecular Lymphatic Sinus

**Figure 3:**
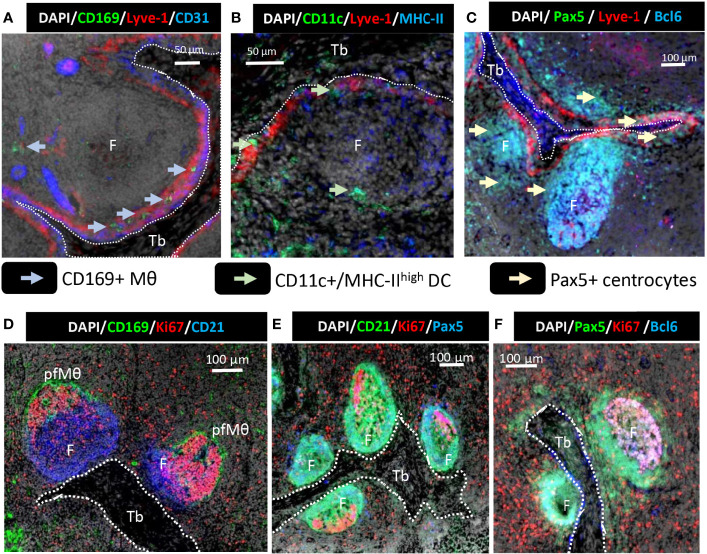
Trabecular lymphatic sinus and B cell follicles relative positioning 10 µm thick sections of a tracheobronchial LN were stained using **(A)** anti-CD169, anti-Lyve-1, anti-CD31 antibodies and DAPI; **(B)** anti-CD11c, anti-Lyve-1, anti-MHC-II and DAPI; **(C)** anti-Pax5, anti-Lyve-1, anti-Bcl6 and DAPI, **(D)** anti-CD169, anti-Ki67, anti-CD21 and DAPI; **(E)** anti- CD21, anti-Ki67, anti-Pax5 and DAPI; **(F)** anti- Pax5, anti-Ki67, anti- Bcl6 and DAPI. F, follicle; Tb, Trabeculae; pfMθ, perifollicular macrophages. Light blue arrow: CD169^pos^ macrophages, light green arrows: CD11c^pos^/MHC-II^high^ dendritic cells, light yellow arrows: centrocytes. **(A, B)**, scale bar 50µm, **(C–F)** scale bar 100µm. Whole LN pictures are individual images from 10x objective acquisitions. Images are representative of 3 LN from different animals.

According to the gross orientation of whole LN images (**Figures 1A, B**), and in agreement with previous work (11, 22), trabeculae support afferent peritrabecular sinuses. Using higher resolution imaging, it could be observed that the endothelium surrounding the trabeculae was composed of two distinct sheets since CD31 and Lyve-1 staining were not superimposed but alongside each other, delimiting a peritrabecular sinus (**Figure 3A**). The floor of this peritrabecular sinus, in contact with the trabeculae, expressed CD31 whereas the ceiling of the peritrabecular sinus, in contact with the B and T cell zones expressed Lyve-1 (**Figure 3A**). This peritrabecular sinus contained CD169^pos^ macrophages (**Figure 3A**, light blue arrows), CD11c^pos^/MHC-II^high^ DC (**Figure 3B**, light green arrows), as well as Pax5^pos^ cells (**Figure 3C**, light yellow arrows), likely recycling centrocytes (29).

Inside follicles, Ki67^pos^/CD21^low^ centroblasts were present on the side surrounded by pfMθ (**Figure 3D**) as previously reported (13). To better identify follicular B cells differentiation steps, Pax5 and BCl6 labeling were carried out. BCl6 is expressed on centroblasts whereas Pax5 is expressed on centroblasts and centrocytes. The use of CD21 labelling with Ki67 and Pax5 revealed that Ki67^neg^/Pax5^pos^ cells were CD21^pos^ (**Figure 3E**) in agreement with centrocytes identity. BCl6 was observed almost exclusively on follicular Ki67^pos^ cells, in agreement with its expression on centroblasts (**Figure 3F**). Bcl6^pos^/Ki67^pos^/Pax5^pos^ centroblasts could be clearly distinguished from BCl6-/Ki67-/Pax5^pos^ centrocytes (**Figure 3F**). The follicles were systematically positioned with the pfMθ and centroblasts (dark zone) at the opposite of the trabeculae.

### Whole LN Vessels Distribution

**Figure 4:**
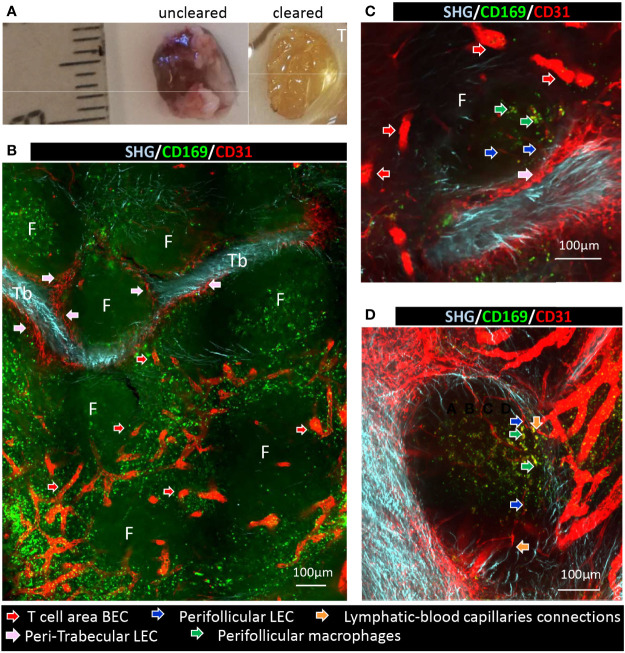
Trabecular sinus, perifollicular sinus and blood vessels interconnections in relation with the perifollicular macrophages. **(A)** Thick slice of tracheobronchial LN were cleared using iDISCO+ and **(B–D)** different area and magnifications of cleared LN slice stained with anti-CD169 and anti-CD31 antibodies. Imaged with multiphoton microscope A1RMP+ with an objective lens 25x and dual excitation at 960 nm and 1040nm. From 960 nm excitation, Second Harmonic generation (SHG) was acquired in blue channel in forward direction and fluorescence of Alexa Fluor 488 was acquired in green channel in backward direction. From 1040 nm excitation, fluorescence of Alexa Fluor 555 was acquired in red channel in backward direction. F, follicle; Tb, Trabeculae. Red arrows: T cell area blood endothelial cells, pink arrows: peri-trabecular lymphatic endothelial cells, dark blue arrows: perifollicular lymphatic endothelial cells, green arrows: perifollicular macrophages, orange arrows: lymphatic-blood capillaries connections. **(A–C)**, scale bar 100µm.

To better apprehend the continuity of the different endothelia, a LN was cleared and stained for CD31 to visualize endothelial cells (**Figure 4A** and **Movie 1**), and for CD169 to localize pfMθ and efferent area occupied by efferent Mθ. Lymphatic and blood endothelia can be differentiated thanks to their different intensity of CD31 staining, LEC being CD31^low^ and BEC CD31^high^. Moreover, biphoton microscopy allowed the second harmonic generation (SHG) imaging to visualize collagenous fibers constituting the trabeculae core. CD31^low^ structures sprouting from SHG-positive collagen-fibers-filled trabeculae were thus unambiguously identified as lymphatic sinuses (**Figure 4B**, pink arrows and **Movie 2**), whereas CD31^high^ vessels were blood vessels (**Figure 4B**, red arrows and **Movie 2**). A 3D reconstruction of **Figure 4A** area allows the visualization of the trabeculae as a flat baggy collagenous area draped in a lymphatic sinus on which stand B cell follicles (**Movie 3**). Thin lymphatic structures were observed sprouting from the trabecular sinus and reaching the perifollicular area (**Figures 4C, D**, blue arrows, movies 4 (confocal imaging) and 5 (biphoton imaging)). Perifollicular Mθ were systematically situated in close proximity with these perifollicular sinuses (**Figures 4C, D**, green arrows). This observation is in agreement with our previous identification of pfMθ as the porcine counterpart of murine SCS Mθ (13) which translocate antigens from the afferent sinus to the B cell follicle in mice (2, 3). In the pfMθ area, continuity between the thin perifollicular sinuses and larger blood capillaries were frequently observed (**Figure 4D**, orange arrows and **Movies 4, 5**).

### LN Blood Vessels

**Figure 5:**
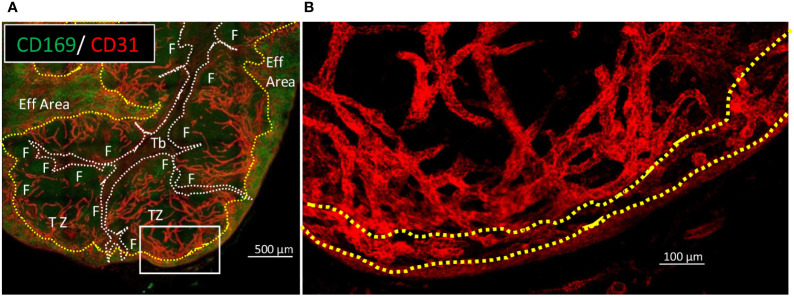
Blood vessels are evenly distributed from B cell follicles neighborhood to the inside of the efferent area. Thick sections of tracheobronchial LN have been cleared with iDISCO+ and stained for CD169 (green) and CD31 (red). **(A)** wide view of CD169 (green) and CD31 (red) staining in order to delimit trabeculae (doted white lines) and efferent area (dashed yellow lines). F, Follicle; Tb, Trabeculae; TZ, T cell zone; Eff Area, efferent area. **(B)** Close up the white rectangle from **(A)**, with depicture of CD31 (red) staining only. Images were acquired with confocal microscope LSM780. **(A)**, Scale bar 500µm. **(B)**, scale bar 100µm Images are representative of 2 LN from one animal.

**Figure 6:**
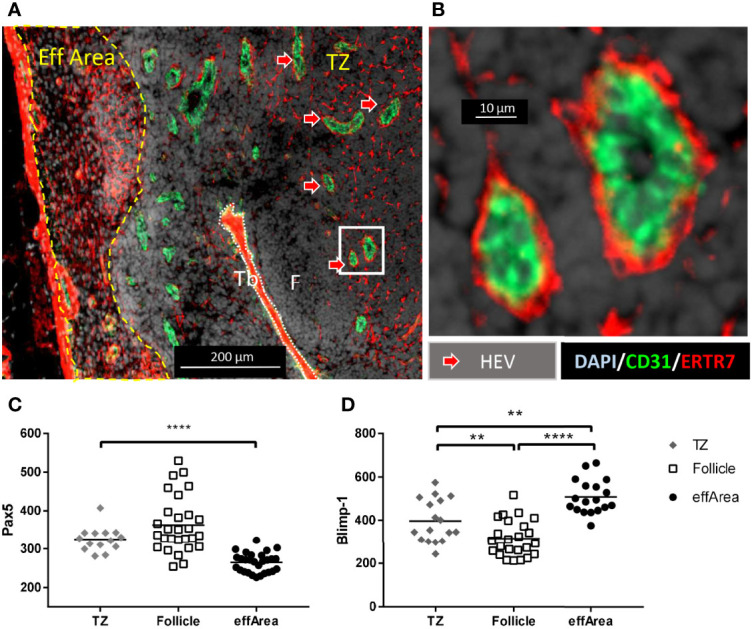
HEV are present in T cell areas, whereas cells presenting mature B cells features are present in the efferent area. 10 µm thick sections of a tracheobronchial LN have been stained for **(A)** Wide view of CD31 (green), ERTR7 (red) and DAPI (blue) staining, **(B)** Close up from the white rectangle from **(A)**. Eff Area, efferent area; TZ, T cell zone; F, follicle; Tb, Trabeculae. **(C)** Pax5 expression on CD21 and CD79α-positive B cells [see **Supplementary Figure 1(A)**], or **(D)** Blimp-1 expression on CD21 and IgM positive B cells [see **Supplementary Figure 1(B)**]. The fluorescence of Pax5 and Blimp-1 were measured using Zen Blue software in different area of the LN: T cell area (TZ), the B cell follicles (Follicle), and the efferent area (effArea). Each symbol represents fluorescence measured on one cell. Since the data were non normally distributed (normality tested using Shapiro-Wilk test) the non-parametric Mann-Whitney test has been chosen. Images are representative of 2 LN from two animals. **(A)** Scale bar 200 µm, **(B)** scale bar 10 µm. ** p<0.01, **** p<0.0001.

Having described the afferent lymphatic structures that might potentially transport free antigens to the B cell follicle, we then investigated the blood transportation circuit allowing the arrival of naïve lymphocytes and the exit of mature, antigen-trained lymphocytes. CD31^high^ structures, corresponding to the veCadh^pos^ vessels observed in **Figure 2A** (red arrows), extended inside the efferent area (**Figures 5A, B** and **Movie 6**). They developed in a network from the efferent area throughout the T cell zones and eventually circled the B cell follicles (**Movie 1** and **Figures 4B, C**). To better understand the entry and exit of naïve and mature B lymphocytes, the localization of HEV along this blood vessel network was investigated. The most used HEV marker is the Peripheral node addressin (PNAd), however we did not identify commercial antibody reacting with porcine PNaD. Another recognized way to identify HEV is their surrounding by fibroblast reticular cells [FRC, for review see (30)], we used the ER-TR7 monoclonal antibody, which stains murine FRC (31) and cross-react with porcine FRC, and used it in combination with anti-CD31. The trabeculae were densely packed with FRC (**Figures 6A, B**), in agreement with FRC’s role in collagen deposit (32). In the TZ and in the efferent area, less dense FRC network was observed, whereas B cell follicles were devoid of FRC. In the TZ, CD31^pos^ endothelial vessels presenting thick walls appeared surrounded by thin FRC sheath (**Figure 6A**), in agreement with an HEV identity.

CD79α is the B-cell antigen receptor complex-associated protein alpha chain which associates with membrane-bound immunoglobulin to form the B-cell antigen receptor (BCR), expressed on naïve and mature B cells (1). We then stained with markers allowing the distinction between naïve and mature B cells in order to identify the LN area where were respectively localized the entry blood vessels, occupied by naïve B cells, and the exit blood vessels occupied by mature B cells. Pax5 has been described as more expressed on naïve than on mature B cells, whereas conversely, Blimp-1 is more expressed on mature than on naïve B cells (1, 33). The expression of Pax5 was measured on CD79α-positive cells in the TZ and the effArea and on CD21-positive cells in the follicles, where centrocytes and centroblasts do not express the BCR (**Supplementary Figure 1A**). B cells in TZ, where HEV are situated (**Figure 6A**) expressed higher levels of Pax5 than B cells from effArea whereas, as expected, follicular B cells expressed contrasted Pax5 levels in agreement with the presence of Pax5^high^ centroblasts and centrocytes and Pax5^low^ plasmablasts (**Figure 6C**). The expression of Blimp-1 was measured on IgM-positive cells and on CD21-positive cells in the follicles, where centrocytes and centroblasts do not express the BCR (**Supplementary Figure 1B**). B cells in TZ expressed low levels of Blimp-1 which are again diminished upon entry of the B cells in the follicle. Then, B cells in the effArea up-regulated Blimp-1, in agreement with the presence of mature B cells in the efferent area (**Figure 6D**).

Thus TZ HEV are in contact with *bona fide* naïve B cells whereas blood vessels in the efferent area are in contact with *bona fide* mature B cells. We thus propose that in agreement with the global orientation of the porcine LN, mature B cells exit from the LN in majority through efferent area blood vessels instead of TZ HEV.

## Discussion

In this work, as previously observed (13), we confirmed the strict orientation of the follicle dark (centroblasts-occupied) and light zones regarding the trabecular sinus. We observed that the trabecular sinus contained *bona fide* CD169^pos^ macrophages and DC as expected for an afferent lymphatic, but also Pax5^pos^ recirculating centrocytes exiting B cell follicle, in agreement with a role of the trabecular sinus as a continuous distribution system from the LN afferent side to the efferent area. Perifollicular Mθ were phenotypically similar to murine SCS Mθ which are involved in the translocation of soluble antigens form the lymphatic afferent sinus to the inside of the follicle. However, pfMθ were not in contact with the afferent peritrabecular sinus since the porcine B cell follicles were systematically oriented with the pfMθ on the side of the follicle in opposition with the trabecullar sinus. We observed here that thin perifollicular lymphatics connect the trabecular sinus with the pfMθ at the opposite side of the follicle. In the perifollicular area, these lymphatics directly connect with blood vessels. Interestingly, lymphatic and blood vessels fusions have been observed *in vitro* in rat mesenteric culture models, leading to Lyve-1 expression decrease on the lymphatic endothelial cells (34), as observed here for the perifollicular lymphatics. We can hypothesize that afferent lymphatic/venules fusion might allow inflammatory cytokines/chemokines transported by afferent lymph from inflamed tissues to reach blood-born immune cells and facilitate their extravasation through the HEV into the inflamed LN, in a similar way to what has been described for collagen/FRC conduits in murine lymph nodes (5, 35).

In human and mouse, Lyve-1 is expressed on medullary LEC (27) whereas in mice Lyve-1 is also expressed on endothelial cells forming the floor of the subcapsular sinus, in contact with SCS Mθ (25). We observed in swine an asymmetric Lyve-1 expression restricted to the ceiling of the peritrabecular afferent sinus, in contact with B and T cell zones. According to the inversion of the porcine LN, this expression appeared similar to what has been observed in mouse. Moreover, like in human and mouse, upon its entry in the efferent area, the peritrabecular sinus loses its asymmetry, and appeared totally Lyve-1^pos^. Interestingly, Lyve-1 is thought to facilitate DC egress from the lymphatic (36), thus its expression on the ceiling of the peritrabecular sinus confirms the similarity between the murine subscapular sinus and the porcine peritrabecular sinus, whereas its expression on both side of the sinus in the efferent area confirms the similarity between porcine, murine and human LN efferent area.

Subscapular sinus EC expressed veCadh, however veCadh is not an exclusive marker of BEC. Indeed, it has been described that human (23, 37) and murine (38) LEC expressed veCadh. Moreover, the porcine subscapular sinus EC were in the continuity of the peritrabecular lymphatic sinus, expressed Lyve-1 and presented lower levels of CD31 than BEC, in agreement with a *bona fide* LEC identity.

**Figure 7:**
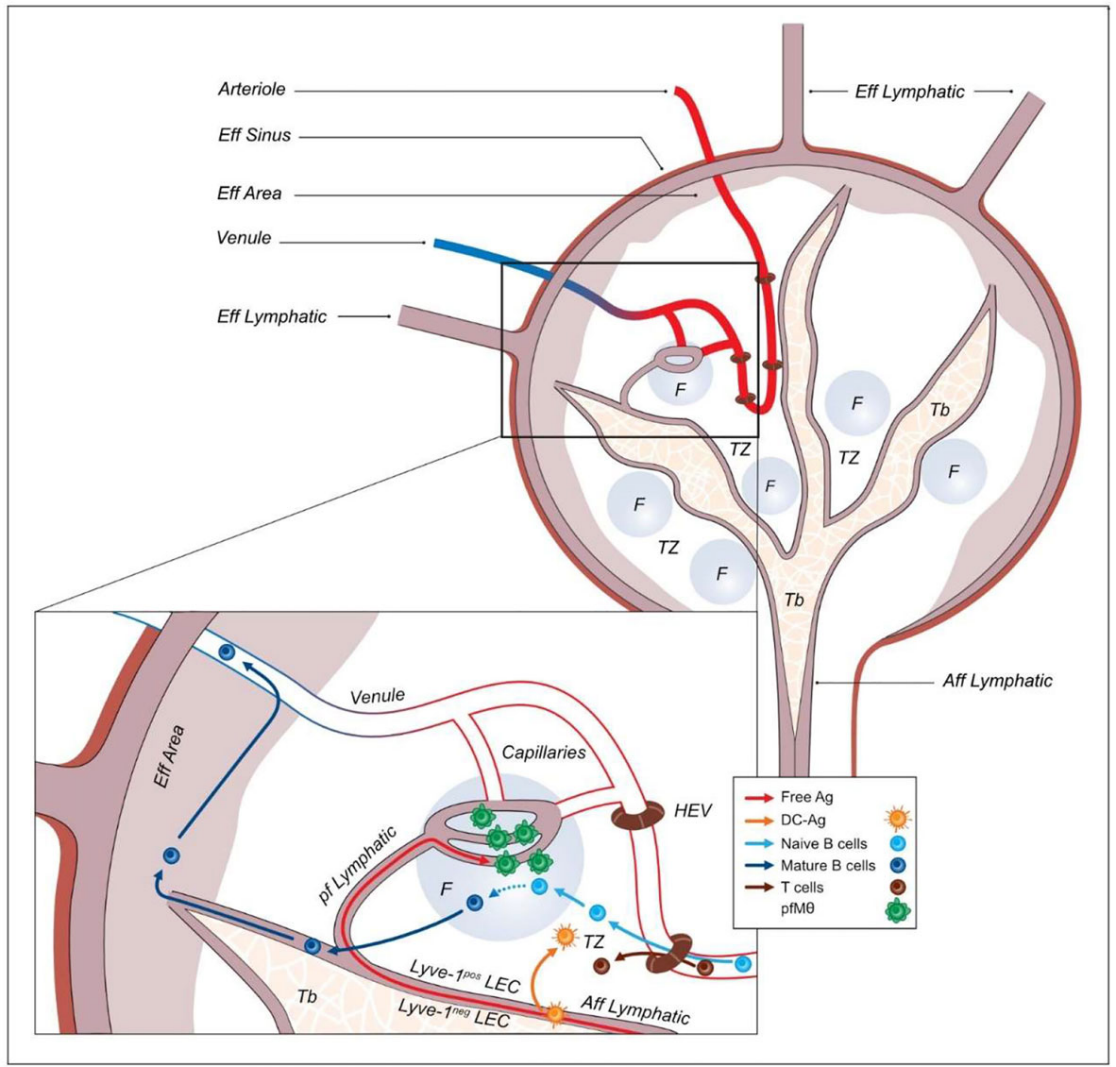
Schematic depicting of the lymphatic and blood flow according to immune cells compartments. Aff Lymphatic, Afferent lymphatic; Tb, Trabeculae; F, follicle; TZ, T cell zone; Eff Area, efferent area; Eff Sinus, efferent sinus; Eff Lymphatic, Efferent lymphatic; HEV, High endothelial venule; LEC, Lymphatic endothelial cells; pf Lymphatic, perifollicular lymphatic; Free Ag, Free antigen; DC-Ag, Antigen transported by DC; pfMθ, perifollicular macrophage.

Interestingly, in swine conversely to mice and human, efferent lymph carries no cell, since activated immune B and T lymphocytes exit the inverted-LN through blood vessels. It was hypothesized for long that this egress was through the HEV (19), bringing up the intriguing conundrum of a common entry and exit way for the naïve and mature lymphocytes. Herein we observed that as previously described, HEV are localized in the TZ and the continuity of HEV bearing vessels leads to the efferent area. The B cells present in the efferent area, are more mature than B cells in the TZ, expressing lower levels of Pax5 and higher levels of Blimp-1. This result is in agreement with Pabst et al. (19) who observed a centrifuge move of B cells from TZ HEV to the LN periphery. We thus propose that in swine, mature B cells exit lymph nodes in the efferent area, without using HEV. It remains to be explored if the portion of blood vessels present in the efferent area harbors specialized features such as the expressions of a peculiar set of addressins that would facilitate the egress of mature B cells. In the inverted pig LN, blood vessels enter and exit the LN throughout the capsule, and not, or at least not exclusively, through the hilum (14). We thus propose here a refined model of the lymphatic and blood circuitry in the inverted porcine LN (**Figure 7** and **Table 2**) that allows a better understanding of the immune cells circulation. Briefly, like in regular human and murine LN, porcine naïve lymphocytes enter the LN through the HEV, situated in the T cell area, whereas free or DC-associated antigens entered through the afferent lymph. The porcine afferent lymphatics are appended on a collagen-composed trabecular structure which crosses the entire LN. DC-associated antigens would reach the T cell area thanks to the asymmetric expression of Lyve-1 on the afferent lymphatic endothelial cells facing the T cell area. Free antigens would reach the B cell follicle through thin perifollicular lymphatics that connect the main peritrabecular lymphatic to the dark zone side of the B cell follicle containing centroblasts. There, pfMθ, the SCS Mθ counterpart of swine (13), control the antigens transfer from the lymphatics to the B cell follicle. Post-follicular B cells may then reach the efferent area and exit the LN through the venules. In the follicle vicinity, perifollicular lymphatics fuse with capillaries, potentially allowing the communication between afferent lymph compounds coming from the drained, inflamed tissues, with blood cells arriving from the HEV, as observed in the murine model (5). We are aware that this model is based on static images. The next step, out of the scope of this work, would be to associate up to date fluorescent microscopy imaging with intra-arterial and intra-lymphatic injections of tracers (5, 14, 15, 19) that would validate our model.

**Table 2:** Comparison of the main murine and porcine LN differences.

		Murine LN	Porcine LN
**Lymph flow**		*Centripetal*	*Centrifugal*
**Afferent lymphatic**	Side in contact with pfMθ	*Floor of the* *scSinus (39)*	*pfLymph*
	Side in contact with TZ	*Floor of the scSinus* ***Lyve-1^pos^*** *(25)*	*Ceiling of the affLymph* ***Lyve-1^pos^***
	Side opposed to TZ	*Ceiling of the scSinus* ***Lyve-1^neg^*** *(25)*	*Floor of the affLymph* ***Lyve-1^neg^***
	Connection with blood	*Collagen conduits connecting scSinus to* *HEV (5, 35)*	*pfLymph connecting to* *post-HEV capillaries*
**B cell follicles**		*Subcapsular/Peripheral*	*Core/Central*
**Efferent area**		*Core/central*	*Subcapsular*
**Mature B cell exit**		*Efferent lymphatic*	*Post-HEV venules*

LN, Lymph Node; pfMθ, perifollicular macrophages (murine subcapsualr sinus macrophages); scSinus, subcapsular sinus; pfLymphatic, perifollicular lymphatics; affLymph, afferent lymphatics; TZ, T cell Zone; HEV, High Endothelial Venule.

By increasing our knowledge on porcine LN endothelial cells this work might allow to proceed to single cell RNAseq of these endothelial cells in order to have a better view of their heterogeneity, and to be able to compare them with recent mouse and human data (26, 40). The pig is more and more recognized as a immunological medical model (41–46), and genetically modified pigs are now developed for direct medical usage such as xenotransplantations (47). Thus a better knowledge of the first steps of the immune response initiation in the inverted porcine LN appears essential. The cellular and molecular processes that determine normal murine or human LN formation are far from being fully deciphered (48). By its oddness, the inverted porcine LN development would deserve a deeper attention since it might offer an original point of view (**Table 2**) permitting to better understand the development of normal LN in healthy and pathological situations. In addition to its functional and developmental implications, the last puzzle of inverted LN is their repartition among the evolutionary tree. In the *Laurasiatheria* superorder, three families of the *Cetarthiodactyles* orders [*Suidae*, *Hippopotamidae* and *Delphinidae* (22, 49)], and one from the *Perissodactyles* orders [*Rhinocerotidae* (50, 51)] present LN inversion. In addition, *Elephantidae* (50, 52), from the *Afrotheria* superorder would also present LN inversion. Except for swine, all the information on inverted LN refereed to 1960 and 1970’s studies. Thus, this field of investigation would greatly benefit of new data taking advantage of last technical developments. Although the study of these various species, among them some endangered, is of great interest, the collect of tissues from these wild, rare, long-lived animals poses strong constraints that would need prolonged coordinated efforts to complete.

## Data Availability Statement

The raw data supporting the conclusions of this article will be made available by the authors, without undue reservation.

## Ethics Statement

The animal study was reviewed and approved by Pays de Loire ethics committee. Authorization no. 2020062915381908/APAFIS 26250v3.

## Author Contributions

LD supervised, acquired and processed the cleared whole LN staining, acquisition and images analysis. LD processed the 3D movies and wrote part of the M&M. ML cut the OCT-frozen samples, proceeded to the HES staining and acquired the 2D bright light and fluorescence images on the slide scanner. CH, DM, CP, and NB proceeded to the immunostaining of LN slides. FJM acquired and processed some cleared whole LN. TL collected and snap-froze the LN. LD, TL, and FM corrected and edited the manuscript, providing thorough discussions and critical manuscript reading. NB supervised the work, designed the experiments, analyzed the images, prepared the figures and wrote the manuscript. All authors contributed to the article and approved the submitted version.

## Funding

APEX, PAnTher, INRAE, Oniris is supported by Pays de la Loire, IBISA and NeurATRIS translational research infrastructure for innovative therapies in NeuroSciences. Biogenouest (the network of technology core facilities in Western France in life sciences and the environment, and by the Conseil Régional des Pays de la Loire). APEX platform is a Center of Excellence Nikon. Reagents and equipment were purchased thanks to the establishment grant obtained by FM from the *Région Pays de la Loire* (RFI Food for tomorrow-Cap aliment).

## Conflict of Interest

The authors declare that the research was conducted in the absence of any commercial or financial relationships that could be construed as a potential conflict of interest.

## Publisher’s Note

All claims expressed in this article are solely those of the authors and do not necessarily represent those of their affiliated organizations, or those of the publisher, the editors and the reviewers. Any product that may be evaluated in this article, or claim that may be made by its manufacturer, is not guaranteed or endorsed by the publisher.

## Supplementary Material

Movie 1 3D reconstruction of iDISCO+ Cleared whole lymph node image with light sheet. Visualization of endothelial cells by CD31 red fluorescent immunolabeling.

Movie 2 Z stack reconstruction of Trabeculae, Lymphatic and blood vessels of iDISCO+ cleared thick LN slice observed from Z_0µm_ to Z_295µm_ by using multiphoton microscopy. Visualization of -Trabeculae (collagen fibers visualized by second harmonic generation (SHG) - cyan); -Lymphatic and blood vessels (CD31 staining – red): lymphatic vessels presented a discontinuous mild red staining and were in close contact with the cyan collagen whereas blood vessels presented a continuous intense red staining; - CD169^pos^ macrophages (CD169 – green). B cell follicles locations can be inferred by dark, round area devoid of collagen and vessels. Perifollicular macrophages are CD169^pos^ macrophages situated in close contact (above or below) with the follicles. Multiphoton imaging. Images were acquired with a Nikon A1RMP+, objective APO LWD 25x 1.10W. Two photon excitation fluorescence was used to image CD169 Alexa Fluor 488 in green channel and CD31 Alexa Fluor 555 in red channel. SHG was acquired in blue channel. Size of image: 1421µmx927µmx300µm, Zstack of 60 images, Zstep 5µm.

Movie 3 3D reconstruction of second harmonic generation (SHG) collagen fibers (trabeculae) and CD31^pos^ blood vessels (red) of iDISCO+ cleared thick LN slice analyzed with multiphoton microscope. Three-dimensional image analysis was performed using GA3 module of NIS software package (5.30.03 Nikon). SHG signal was thresholded and segmented. SHG^pos^: objects binarized were colorized to facilitate the observation of 3D organization of Trabeculae collagen fibers (Size range of total objects SHG^pos^: 5 to 84140301 µm3). The biggest continuous object with SHG^pos^ signal representing the Trabeculae was depicted in blue surrounded with a white line to improve the visualization of the trabeculae 3D organization. The lower discrete SHG^pos^ objects were depicted in green, yellow, pink, purple according to their size and their distribution. CD31^pos^ endothelial cells are in red. Size of image 1400µmx1138µmx675µm.

Movie 4 Z-stacks reconstruction of Lymphatic and blood vessels (CD31 staining – red) of iDISCO+ cleared thick LN slice analyzed with confocal microscope. Lymphatic vessels presented a discontinuous mild red staining whereas blood vessels presented a continuous intense red staining; CD169^pos^ macrophages (CD169 – green). B cell follicles locations can be inferred by dark, round area devoid of vessels. Perifollicular macrophages are CD169^pos^ macrophages situated in close contact (above or below) with the follicles. Size of image 500µmx500µmx200µm, Zstack of 100 images, Zstep 2µm.

Movie 5 Z-stacks reconstruction of Trabeculae (collagen fibers visualized by second harmonic generation - cyan), lymphatic and blood vessels (CD31 staining – red) of iDISCO+ cleared thick LN slice analyzed with multiphoton microscope. Lymphatic vessels presented a discontinuous mild red staining and were in close contact with the cyan collagen whereas blood vessels presented a continuous intense red staining; CD169^pos^ macrophages (CD169 – green). B cell follicles locations can be inferred by dark, round area devoid of collagen and vessels. Perifollicular macrophages are CD169^pos^ macrophages situated in close contact (above or below) with the follicles. Size of image 586µmµmx628µmx220µm, Zstack of 45 images, Zstep 5µm.

Movie 6 3D reconstruction of lymph node vasculature (CD31 staining) from follicles (on the right at the start of the movie) to the efferent area (at the left at the start of the movie). Multiphoton imaging, scale at left with depth coded maximum intensity projection (size image 1438µmx1438µmx390µm).

## References

[1] Klein U Dalla-Favera R. Germinal Centres: Role in B-Cell Physiology and Malignancy. Nat Rev Immunol (2008) 8:22–33. doi: 10.1038/nri2217 18097447

[2] Phan TG Green JA Xu Y Cyster JG. Immune Complex Relay by Subcapsular Sinus Macrophages and Noncognate B Cells Drives Antibody Affinity Maturation. Nat Immunol (2009) 10:786–96. doi: 10.1038/ni.1745 PMC2776777 19503106

[3] Phan TG Grigorova I Okada T Cyster JG. Subcapsular Encounter and Complement-Dependent Transport of Immune Complexes by Lymph Node B Cells. Nat Immunol (2007) 8:992–1000. doi: 10.1038/ni1494 17660822

[4] Novkovic M Onder L Cupovic J Abe J Bomze D. Topological Small-World Organization of the Fibroblastic Reticular Cell Network Determines Lymph Node Functionality. PloS Biol (2016) 14:1–20. doi: 10.1371/journal.pbio.1002515 PMC4945005 27415420

[5] Sixt M Kanazawa N Selg M Samson T Roos G Reinhardt DP. The Conduit System Transports Soluble Antigens From the Afferent Lymph to Resident Dendritic Cells in the T Cell Area of the Lymph Node. Immunity (2005) 22:19–29. doi: 10.1016/j.immuni.2004.11.013 15664156

[6] Roozendaal R Mebius RE Kraal G. The Conduit System of the Lymph Node. Int Immunol (2008) 20:1483–7. doi: 10.1093/intimm/dxn110 18824503

[7] Gaya M Castello A Montaner B Rogers N Reis e Sousa C Bruckbauer A. Inflammation-Induced Disruption of SCS Macrophages Impairs B Cell Responses to Secondary Infection. Science (2015) 347:667. doi: 10.1126/science.aaa1300 25657250

[8] Sinkora M Butler JE Lager KM Potockova H Sinkorova J. The Comparative Profile of Lymphoid Cells and the T and B Cell Spectratype of Germ-Free Piglets Infected With Viruses SIV, PRRSV or PCV2. Vet Res (2014) 45:1–18. doi: 10.1186/s13567-014-0091-x 25186625 PMC4156959

[9] Hunt AC. Micro-Anatomy of the Lymph Nodes of the Pig. Br J Exp Pathol (1968) 49:338–9. PMC2093832 5676443

[10] Mcfarlin DE Binns RM W. Lymph node function and lymphocyte circulation in the pig. Adv Exp Med Biol (1973) 29:87–93. doi: 10.1007/978-1-4615-9017-0_13 4137044

[11] Bowes MA Kenny AJ. Endopeptidase-24.11 in Pig Lymph Nodes. Purification and Immunocytochemical Localization in Reticular Cells. Biochem J (1986) 236:801–10. doi: 10.1042/bj2360801 PMC1146913 3539105

[12] Pabst R Binns RM Licence ST. Surface Markers on Lymphocytes Leaving Pig Lymph Nodes. Immunology (1985) 56:301–6. PMC1453694 3876982

[13] Bordet E Frétaud M Crisci E Bouguyon E Rault S Pezant J. Macrophage-B Cell Interactions in the Inverted Porcine Lymph Node and Their Response to Porcine Reproductive and Respiratory Syndrome Virus. Front Immunol (2019) 10:953. doi: 10.3389/fimmu.2019.00953 31130951 PMC6510060

[14] Splading H Heath T. Blood Vessels of Lymph Nodes in the Pig. Res Vet Sci (1986) 41:196–9. doi: 10.1016/S0034-5288(18)30598-8 3775110

[15] Spalding H Heath T. Pathways of Lymph Flow Through Superficial Inguinal Lymph Nodes in the Pig. Anat Rec (1987) 217:188–95. doi: 10.1002/ar.1092170211 3578836

[16] Spalding HJ Heath TJ. Inguinal Lymph Centre in the Pig. (1989), 43–54. PMC1256738 2621146

[17] Binns RM Pabst R Licence ST. Lymphocyte Emigration From Lymph Nodes by Blood in the Pig and Efferent Lymph in the Sheep. Immunology (1985) 54:105–11. PMC1454856 3972428

[18] Pabst R Binns RM. *In Vivo* Labelling of the Spleen and Mesenteric Lymph Nodes With Fluorescein Isothiocyanate for Lymphocyte Migration Studies. Immunology (1981) 44:321–9. PMC1555225 6795108

[19] Pabst R Geisler R. The Route of Migration of Lymphocytes From Blood to Spleen and Mesenteric Lymph Nodes in the Pig. Cell Tissue Res (1981) 221:361–70. doi: 10.1007/BF00216740 7307059

[20] Renier N Adams EL Kirst C Wu Z Azevedo R Kohl J. Mapping of Brain Activity by Automated Volume Analysis of Immediate Early Genes. Cell (2016) 165:1789–802. doi: 10.1016/j.cell.2016.05.007 PMC4912438 27238021

[21] Pichon J Ledevin M Larcher T Jamme F Rouger K Dubreil L. Label-Free 3D Characterization of Cardiac Fibrosis in Muscular Dystrophy Using SHG Imaging of Cleared Tissue. Biol Cell (2022) 144:1–13. doi: 10.1111/boc.202100056 34964145

[22] Binns RM. Organisation of the Lymphoreticular System and Lymphocyte Markers in the Pig. Vet Immunol Immunopathol (1982) 3:95–146. doi: 10.1016/0165-2427(82)90033-2 7048722

[23] Kriehuber E Breiteneder-Geleff S Groeger M Soleiman A Schoppmann SF Stingl G. Isolation and Characterization of Dermal Lymphatic and Blood Endothelial Cells Reveal Stable and Functionally Specialized Cell Lineages. J Exp Med (2001) 194:797–808. doi: 10.1084/jem.194.6.797 11560995 PMC2195953

[24] Pfeiffer F Kumar V Butz S Vestweber D Imhof BA Stein JV. Distinct Molecular Composition of Blood and Lymphatic Vascular Endothelial Cell Junctions Establishes Specific Functional Barriers Within the Peripheral Lymph Node. Eur J Immunol (2008) 38:2142–55. doi: 10.1002/eji.200838140 18629939

[25] Ulvmar MH Werth K Braun A Kelay P Hub E Eller K. The Atypical Chemokine Receptor CCRL1 Shapes Functional CCL21 Gradients in Lymph Nodes. Nat Immunol (2014) 15:623–30. doi: 10.1038/ni.2889 24813163

[26] Jalkanen S Salmi M. Lymphatic Endothelial Cells of the Lymph Node. Nat Rev Immunol (2020) 20:566–78. doi: 10.1038/s41577-020-0281-x 32094869

[27] Takeda A Hollmén M Dermadi D Pan J Brulois KF Kaukonen R. Single-Cell Survey of Human Lymphatics Unveils Marked Endothelial Cell Heterogeneity and Mechanisms of Homing for Neutrophils. Immunity (2019) 51:561–572.e5. doi: 10.1016/j.immuni.2019.06.027 31402260

[28] Xiang M Grosso RA Takeda A Pan J Bekkhus T Brulois K. A Single-Cell Transcriptional Roadmap of the Mouse and Human Lymph Node Lymphatic Vasculature. Front Cardiovasc Med (2020) 7:52. doi: 10.3389/fcvm.2020.00052 32426372 PMC7204639

[29] Kepler TB Perelson AS. Cyclic Re-Entry of Germinal Center B Cells and the Efficiency of Affinity Maturation. Immunol Today (1993) 14:412–5. doi: 10.1016/0167-5699(93)90145-B 8397781

[30] Ager A. High Endothelial Venules and Other Blood Vessels: Critical Regulators of Lymphoid Organ Development and Function. Front Immunol (2017) 8:45. doi: 10.3389/fimmu.2017.00045 28217126 PMC5289948

[31] Vliet EV Melis M Ewijk WV Wet V. Monoclonal Antibodies to Stromal Cell Types of the Mouse Thymus. Eur J Immunol (1984) 14:524–9. doi: 10.1002/eji.1830140608 6734714

[32] Martinez VG Pankova V Krasny L Huang PH Tape CJ Acton SE. Fibroblastic Reticular Cells Control Conduit Matrix Deposition During Lymph Node Expansion. CellReports (2019) 29:2810–2822.e5. doi: 10.1016/j.celrep.2019.10.103 PMC6899512 31775047

[33] Nutt SL Fairfax KA Kallies A. BLIMP1 Guides the Fate of Effector B and T Cells. Nat Rev Immunol (2007) 7:923–7. doi: 10.1038/nri2204 17965637

[34] Azimi M Motherwell J Hodges N Rittenhouse G Majbour D Porvasnik S. Lymphatic-To-Blood Vessel Transition in Adult Microvascular Networks: A Discovery Made Possible by a Top-Down Approach to Biomimetic Model Development. Microcirculation (2020) 27:e12595. doi: 10.1111/micc.12595 31584728 PMC7033030

[35] Gretz JE Norbury CC Anderson AO Proudfoot AE Shaw S. Lymph-Borne Chemokines and Other Low Molecular Weight Molecules Reach High Endothelial Venules *via* Specialized Conduits While a Functional Barrier Limits Access to the Lymphocyte Microenvironments in Lymph Node Cortex. J Exp Med (2000) 192:1425–40. doi: 10.1084/JEM.192.10.1425 PMC2193184 11085745

[36] Johnson LA Banerji S Lawrance W Gileadi U Prota G Holder KA. Dendritic Cells Enter Lymph Vessels by Hyaluronan-Mediated Docking to the Endothelial Receptor LYVE-1. Nat Immunol (2017) 18:762–70. doi: 10.1038/ni.3750 28504698

[37] Park SM Angel CE McIntosh JD Mansell CM Chen CJJ Cebon J. Mapping the Distinctive Populations of Lymphatic Endothelial Cells in Different Zones of Human Lymph Nodes. PloS One (2014) 9:1–10. doi: 10.1371/journal.pone.0094781 PMC3986404 24733110

[38] Asano K Nabeyama A Miyake Y Qiu CH Kurita A Tomura M. CD169-Positive Macrophages Dominate Antitumor Immunity by Crosspresenting Dead Cell-Associated Antigens. Immunity (2011) 34:85–95. doi: 10.1016/j.immuni.2010.12.011 21194983

[39] Bellomo A Gentek R Bajénoff M Baratin M. Lymph Node Macrophages: Scavengers, Immune Sentinels and Trophic Effectors. Cell Immunol (2018) 330:168–74. doi: 10.1016/j.cellimm.2018.01.010 29397903

[40] Leach SM Gibbings SL Tewari AD Atif SM Vestal B Danhorn T. Human and Mouse Transcriptome Profiling Identifies Cross-Species Homology in Pulmonary and Lymph Node Mononuclear Phagocytes. Cell Rep (2020) 33:108337. doi: 10.1016/j.celrep.2020.108337 33147458 PMC7673261

[41] Bertho N Meurens F. The Pig as a Medical Model for Acquired Respiratory Diseases and Dysfunctions: An Immunological Perspective. Mol Immunol (2021) 135:254–67. doi: 10.1016/j.molimm.2021.03.014 33933817

[42] Gerdts V Wilson HL Meurens F Van den Hurk S van DL Wilson D Walker S. Large Animal Models for Vaccine Development and Testing. ILAR J (2015) 56:53–62. doi: 10.1093/ilar/ilv009 25991698

[43] Käser T Renois F Wilson HL Cnudde T Gerdts V Dillon JAR. Contribution of the Swine Model in the Study of Human Sexually Transmitted Infections. Infect Genet Evol (2018) 66:346–60. doi: 10.1016/j.meegid.2017.11.022 29175001

[44] Lunney JK Van goor A Walker K Hailstock T Franklin J Dai C. Importance of the Pig as a Human Biomedical Model. Sci Transl Med (2021) 13. doi: 10.1126/scitranslmed.abd5758 34818055

[45] Meurens F Summerfield A Nauwynck H Saif L Gerdts V. The Pig: A Model for Human Infectious Diseases. Trends Microbiol (2012) 20:50–7. doi: 10.1016/j.tim.2011.11.002 PMC7173122 22153753

[46] Pabst R. The Pig as a Model for Immunology Research. Cell Tissue Res (2020) 380:287–304. doi: 10.1007/s00441-020-03206-9 32356014 PMC7223737

[47] Reardon S. First Pig-to-Human Heart Transplant: What Can Scientists Learn? Nature (2022) 601:305–6. doi: 10.12968/prma.2021.31.4.8 35031782

[48] Onder L Mörbe U Pikor N Novkovic M Cheng H-W Hehlgans T. Lymphatic Endothelial Cells Control Initiation of Lymph Node Organogenesis. Immunity (2017) 47:80–92.e4. doi: 10.1016/J.IMMUNI.2017.05.008 28709801

[49] Moskov M Schiwatschewa T Bonev S. Comparative Histological Study of Lymph Nodes in Mammals. Lymph Nodes of the Dolphin. Anat Anz (1969) 124:49–67. 5814926

[50] Cave AJE Aumonier FJ. Elephant and Rhinoceros Lymph-Node Histology. J R Microsc Soc (1962) 80:209–14. doi: 10.1111/j.1365-2818.1962.tb00486.x

[51] Cave AJE Aumonier FJ. Lymph Node Structure in the Sumatran Rhinoceros. J R Microsc Soc (1962) 81:73–7. doi: 10.1111/j.1365-2818.1962.tb02071.x

[52] Cave BAJE Aumonier FJ. Lymph Node Structure in an Asiatic Elephant. J R Microsc Soc (1964) 82:251–5. doi: 10.1111/j.1365-2818.1964.tb04479.x 14315863

