# Supplementary material for: The Internal Conduit System of the Swine Inverted Lymph Node
Source: Front Immunol. 2022 Jun 6;13:869384. doi: 10.3389/fimmu.2022.869384 (PMC9207403; doi:10.3389/fimmu.2022.869384)
Supplement: Supplementary file 1 [file DataSheet_1.pdf]

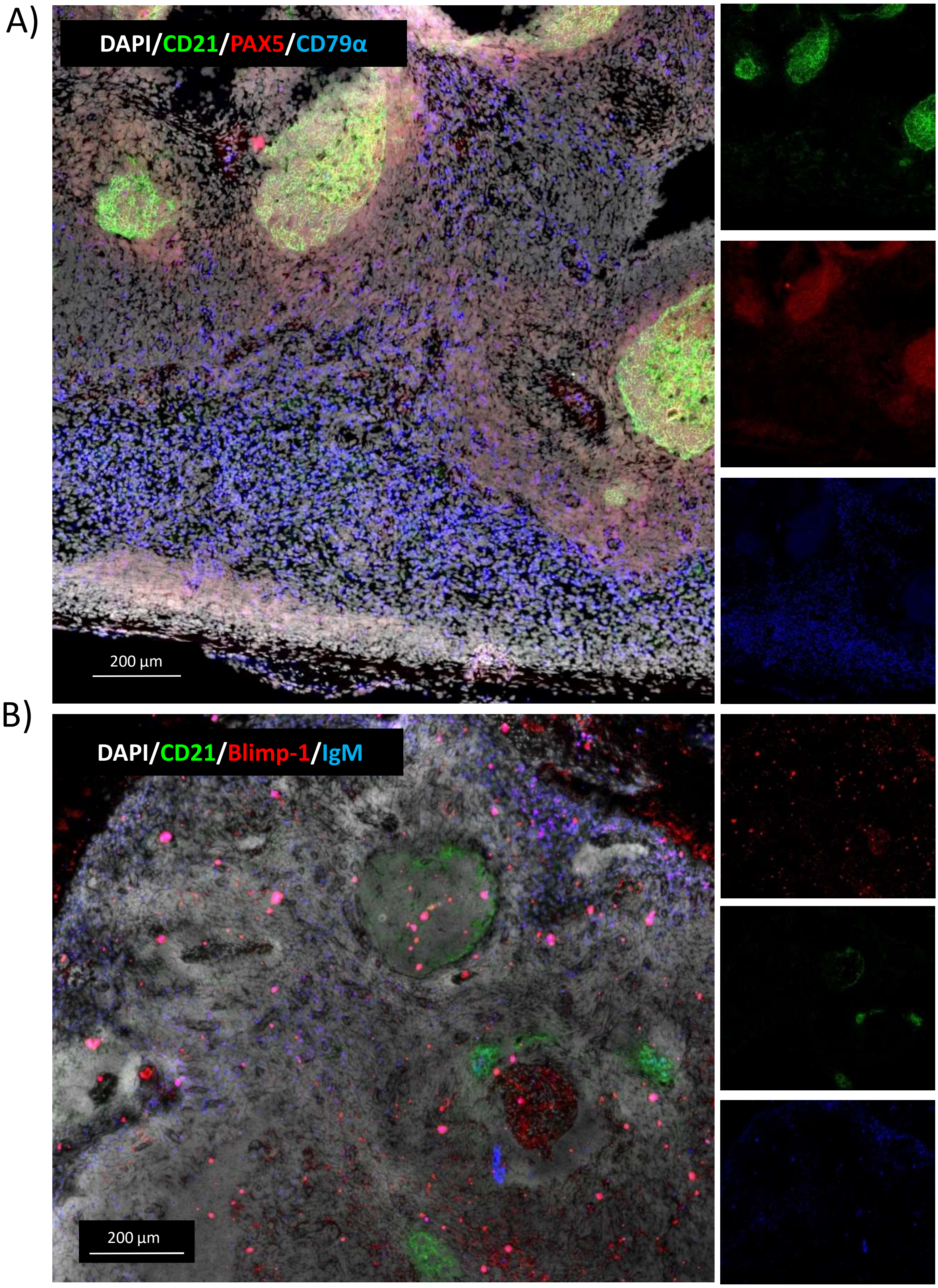

Supp Fig. 1: T cell zones are occupied by naïve, Pax5+ B cells whereas efferent area are occupied by mature, Blimp-1+ B cells. These slices were used for Figure 6 B) and C) Pax5 and Blimp-1 expressions on respectively CD21 or CD79α positive B cells and CD21 or IgM positive B cells .
